# Sickle cell disease in Sri Lanka: clinical and molecular basis and the unanswered questions about disease severity

**DOI:** 10.1186/s13023-020-01458-w

**Published:** 2020-07-06

**Authors:** Thamal Darshana, Dayananda Bandara, Upul Nawarathne, Udaya de Silva, Yasinta Costa, Kalavitigoda Pushpakumara, Sumithra Pathirage, Seuwandi Basnayake, Chamila Epa, Pradeepa Dilrukshi, Maheshaka Wijayawardena, Angela A. Anthony, Rexan Rodrigo, Aresha Manamperi, Frances Smith, Angela Allen, Stephan Menzel, David Rees, Anuja Premawardhena

**Affiliations:** 1 Department of Medical Laboratory Sciences, University of Sri Jayewardenepura, Nugegoda, Sri Lanka; 2 National Thalassaemia Centre, Teaching Hospital, Kurunegala, Sri Lanka; 3 Department of Pediatrics, Teaching Hospital, Kurunegala, Sri Lanka; 4 Thalassaemia Unit, Teaching Hospital, Anuradhapura, Sri Lanka; 5 Department of Haematology, Teaching Hospital, Ragama, Sri Lanka; 6 Department of Pediatrics, District General Hospital, Matara, Sri Lanka; 7 Department of Pediatrics, District General Hospital, Hambantota, Sri Lanka; 8 Department of Haematology, District General Hospital, Monaragala, Sri Lanka; 9 Department of Haematology, Teaching Hospital, Batticaloa, Sri Lanka; 10 Department of Haematology, District General Hospital, Ampara, Sri Lanka; 11 Department of Pediatrics, District General Hospital, Ampara, Sri Lanka; 12 Department of Clinical Sciences, Eastern University, Batticaloa, Sri Lanka; 13 Thalassaemia Adult and Adolescent Care Centre, Teaching Hospital, Ragama, Sri Lanka; 14 Molecular Medicine Unit, University of Kelaniya, Ragama, Sri Lanka; 15 Molecular Pathology Department, Viapath at King’s College Hospital, London, UK; 16 Weatherall Institute of Molecular Medicine, University of Oxford, Oxford, UK; 17 School of Cancer and Pharmaceutical Sciences, The Rayne Institute, King’s College London, London, UK; 18 Department of Haematological Medicine, King’s College Hospital, London, UK; 19 Department of Medicine, University of Kelaniya, Ragama, Sri Lanka

**Keywords:** Sickle cell, Sri Lanka, Genetic, Clinical, Severity

## Abstract

**Background:**

Though case reports and limited case series of Sickle cell disease in Sri Lanka have been reported previously, no attempt has been made hitherto to undertake a comprehensive genotypic-phenotypic analysis of this “rare” group of patients.

**Results:**

All accessible Sickle cell disease patients, totaling 60, including, 51 Sickle β-thalassaemia and 9 homozygous sickle patients were enrolled from seven thalassaemia treatment centres between December 2016–March 2019. The majority of patients were of Sinhalese ethnicity (*n* = 52, 86.67%). Geographically, two prominent clusters were identified and the distribution of Sickle haemoglobin in the island contrasted markedly with the other haemoglobinopathies. 3/ 9 homozygous sickle patients and 3/ 51 Sickle β-thalassaemia patients were receiving regular transfusion. Joint pain was the commonest clinical symptom among all sickle cell disease patients (*n* = 39, 65.0%). Dactylitis was significantly more common in homozygous sickle patients compared with the Sickle β-thalassaemia groups (*p* 0.027). Two genetic backgrounds sickle mutation were identified namely, Arab Indian and Benin. Among the regulators of Foetal hemoglobin in Sickle patients of the present study rs1427407 G > T seemed to be the most prominent modifier, with a significant association with Foetal haemoglobin levels (*p* 0.04).

**Conclusions:**

Overall, the clinical course of the Asian version of Sickle cell disease in Sri Lanka appears to be milder than that described in India.

## Background

Sickle Cell Disease (SCD) is the collective term for a group of inherited disorders characterized by mutations in the gene encoding the β-haemoglobin subunit (HBB). The prevalence of the disease is high in sub-Saharan Africa, Middle East, India, Jamaica and Brazil [1]. Sri Lanka is a multi-ethnic country with a population of 20.4 million, comprised of Sinhalese (74.9%), Tamils (15.2%), Moors (9.3%) and several other minor groups [2].

Sickle haemoglobin (Hb S) was first reported in the country among Sinhalese in 1962 in the Eastern province of the country [3]. Even though Sri Lanka is geographically adjacent to India, where the prevalence of Hb S is high, particularly among tribal populations, the prevalence of Hb S in Sri Lanka is lower and is confined mainly to coastal areas [4]. No detailed descriptions of SCD in Sri Lanka are available in the literature, although there are several reports for some sporadic cases of SCD, including homozygous sickle cell anaemia (Hb SS), sickle-β thalassaemia (SBT) and Hb SD disease [5–10]. Currently, SCD patients in Sri Lanka are typically treated in either thalassaemia centres or general paediatric or medical wards. A recent island-wide hospital based epidemiological survey of haemoglobinopathies identified 1774 patients with a haemoglobinopathy. 51 of whom were sickle patients (un specified SCD), confirming that SCD is uncommon in Sri Lanka (2.8%) (51/1774). The same survey identified significant inconsistencies in care of SCD patients between centres [11]. Genetic information including haplotype analysis for SCD in Sri Lanka is scarce, and has been reported for a single patient with SBT [12] only. Hence, in the present study we intend to describe the clinical picture of SCD patients in Sri Lanka, analyze its molecular basis, including the effects of genetic modifiers on the phenotype.

## Methods

### Study design and population

We conducted a cross-sectional study between December 2016 and March 2019 recruiting patients previously diagnosed with SCD from seven thalassaemia centres in Sri Lanka. The thalassaemia centres were located in the districts of Mahara, Kurunegala, Anuradhapura, Hambantota, Monaragala, Ampara and Batticaloa. All SCD patients were eligible for the study and there were no exclusion criteria. All patients were examined by the study physician and clinical details were obtained using a pre-tested interviewer-administered questionnaire. Ethical approval for the study was obtained from Faculty of Medicine, University of Kelaniya, Sri Lanka (P/01/01/2016). Informed written consents form adult SCD patients and assents from the parents of the participating SCD children were obtained before enrollment for the present study.

### Haematological and haemoglobin analyses

A Five ml venous blood sample was collected into EDTA (Ethylenediaminetetraacetic acid) from each participant. Routine haematological measurements were conducted using a Coulter counter Ac•T 5diff OV (Beckman Coulter, Inc., Brea, California, United States). Haemoglobin phenotype was determined by capillary electrophoresis (CE) using Capillarys 2 flex piercing analyzer (Sebia, France). DNA for genetic analyses was extracted by QIAamp DNA Blood Mini Kit (Qiagen, Hilden, Germany) and stored at − 20 °C until further use.

### Basic genetic analyses

Classical β- globin haplotyping was performed. Six regions around and within the β globin gene cluster were amplified by the polymerase chain reaction (PCR), using primers from Integrated DNA Technologies, Inc., Iowa, United States. Primer sequences were those referenced by [13]. PCR products of each patient were treated with appropriate restriction enzymes (from Thermofisher scientific) according to manufactures instructions and the resulting fragments were separated on 2% agarose gel. Bands were visualized and photographed by UVP BioDoc-It® Imaging System. Six polymorphic restriction sites were studied; 5′ to ε gene by Hind II, 5' to Gγ gene by Xmn I, within IVS 2 of the Gγ and Aγ genes by Hind III, 3′ to ψβ by Hind II, and IVS 2 of the β gene by Ava II. When the Restriction Fragment Length Polymorphism (RFLP) pattern was heterozygous, the sickle haplotype was determined based on the assumption that common sickle haplotypes were present [14]. Common α+ globin gene deletions (3.7 and 4.2 kb) were studied by multiplex GAP polymerase chain reaction [15]. Beta- thalassaemia mutations of the SBT patients were determined by Amplification Refractory Mutation System (ARMS) [16].

### Sequencing analyses of Hb SS patients

New generation sequencing (NGS) was done using a customized panel which sequenced 5 regions of the genome of all the Hb SS patients reported in study including; Chromosome 2 (hg 19 Grch build 37) - chr2:60,575,685 - 60,753,050, Chromosome 6 (hg 19 Grch build 37) - chr6:135,281,347 - 135,540,835, Chromosome 11 (hg 19 Grch build 37) - chr11:3,779,641 - 7,224,114, Chromosome 16 (hg 19 Grch build 37) - chr16: 575,307- 2,619,179 and Chromosome X (hg 19 GrCh build 37) - chrX:11,253,922-11,377,717 using Illumina platform (Illumina Miseq). Variations found were annotated with Integrative Genomic Viewer version 2.6 (Broad Institute) using GRCh37 - hg19 - Genome – Assembly by NCBI (National Centre for bio-informatics) as the reference sequence.

### Genotyping of Foetal Haemoglobin (Hb F) modifiers among SCD patients

Four known Hb F modifiers (rs1427407 and rs6545816 in BCL11A, rs66650371 in HMIP-2A and rs9402686 in HMIP-2B) were genotyped by Taqman assay real time PCR using Viia 7 Applied Biosystems. One Hb F modifier (rs7482144 in Xmn1-HBG2) was genotyped by RFLP. These Hb F markers were selected based on their positive association with Hb F levels in SCD patients suggested by several studies [17–19].

## Results

### Basic demographic data

**Fig. 1:**
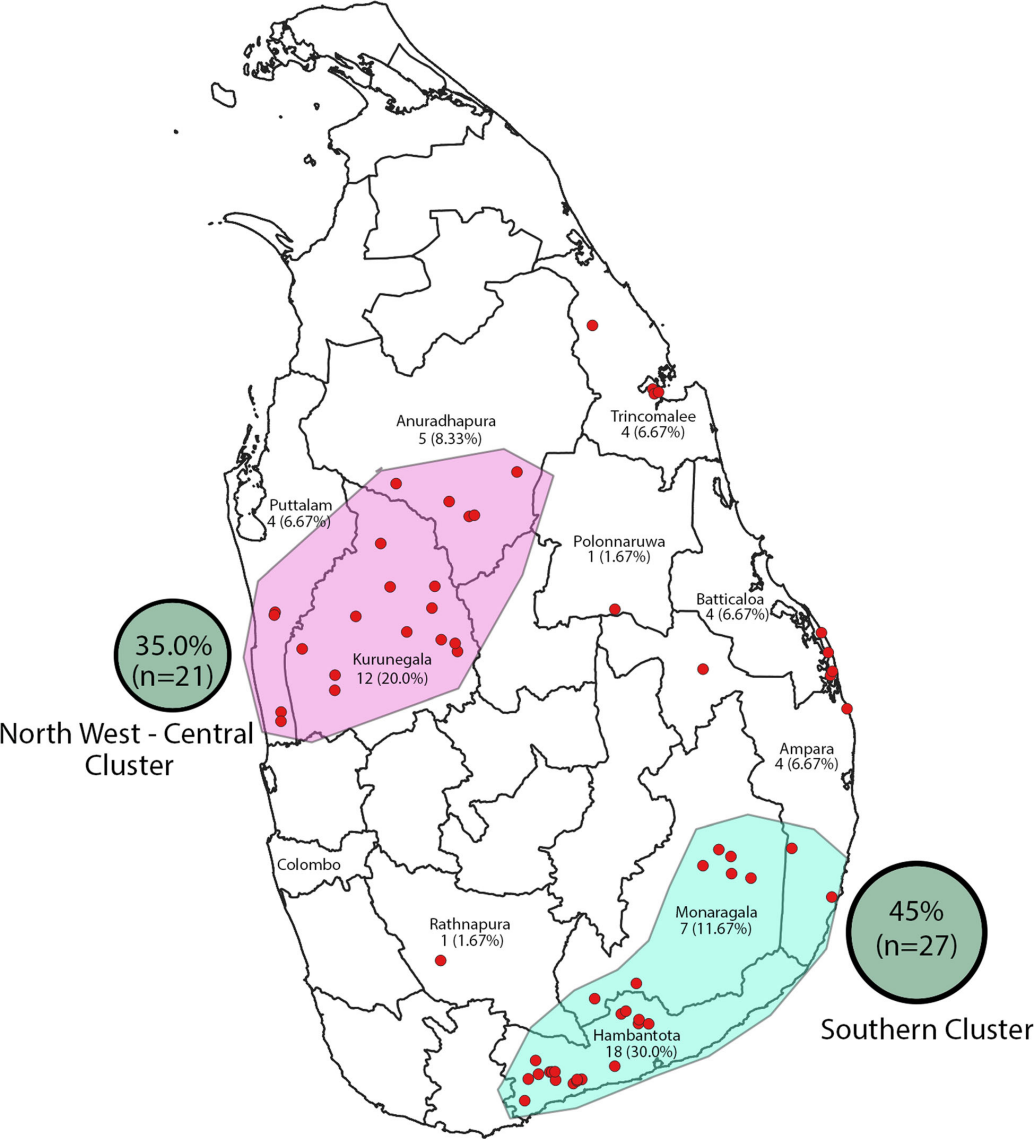
Locations from which SCD patients were reported in present study (each dot represents one patient)

Between December 2016 and March 2019, 60 SCD patients were recruited for the study. Fifty-one patients (51/60; 85%) were SBT patients and 9 (9/60; 15%) were homozygous Hb SS patients. Homozygosity was confirmed in all 9 patients by typing the sickle mutation rs334 (T > A) at chr11:5227002 (GRCh38.p12) by NGS. There were 30 male and 30 female participants. Thirty-seven (72.55%) SBT patients had IVS 1–5 (G → C) mutation, 11(21.57%) had IVS 1–1 (G → A) mutation, 2 had CD-16 mutation and one SBT patient had CD 41/42 mutation. Even though IVS 1–5 (G → C) clinically behaves as an β^0^ mutation for all practical purposes SBT patients with IVS 1–5 (G → C) was classified separately as severe Hb S/β+ as it is known to produce some Hb A (*n* = 37) [20–23]. SBT patients identified with other β mutations were all unquestionably severe mutations and were classified as Hb S/β^0^ type (*n* = 14). SCD patients in the present study were living in 10 out of the 25 districts of Sri Lanka. Geographically, two prominent patient clusters were noted and the Southern cluster comprising Hambantota and Monaragala districts accounting for 27 (45.0%) SCD patients was the dominant cluster (Fig. 1). The SCD patients comprised of three ethnic groups; 52 (86.67%) were Sinhalese, 5 (8.33%) were Moors and 3 (5.0%) were Tamils. Non-parametric statistical methods were used since data were not normally distributed.

### Haematological data

Basic haematological parameters of those who had not received a blood transfusion in the three months prior to blood sampling are summarized in Table 1.

**Table 1:** Haematological parameters of Hb SS, Hb S/β+ severe and Hb S/β^0^ type patients

Parameter	Hb SS			Hb S/β+ (severe) (*n* = 31)			Hb S/β^0^ (*n* = 11)			*p*-value
	Mean (SD)			Mean (SD)			Mean (SD)			
	Male (*n* = 4)	Female (*n* = 2)	Total	Male (*n* = 16)	Female (*n* = 15)	Total	Male (*n* = 5)	Female (*n* = 6)	Total	
**Hb (g/dl)** (13.0–18.0 – Male) (11.5–16.5 Female)	8.4 (1.1)	8.7 (0.2)	8.5 (0.9)	8.3 (1.0)	8.3 (1.0)	8.3 (1.0)	8.0 (0.8)	8.3 (0.6)	8.1 (0.7)	0.787
**Hb A2 (%)** (1.5–3.2%)	1.7 (0.4)	1.4 (0.5)	**1.6 (0.4)**	4.4 (0.6)	4.2 (0.5)	**4.3 (0.5)**	4.9 (0.2)	4.4 (0.6)	**4.6 (0.5)**	< 0.000*
**Hb F (%)** (< 1.0%)	20.6 (1.8)	31.9 (0.5)	24.4 (6.0)	23.2 (6.4)	24.3 (6.4)	23.7 (6.4)	19.1 (5.7)	22.9 (8.7)	21.2 (7.4)	0.514
**MCV (fl)** (80–100)	84.2 (4.3)	85.0 (9.9)	**84.5 (5.6)**	69.3 (4.6)	68.2 (3.9)	**68.8 (4.3)**	67.8 (3.3)	69.2 (4.1)	**68.6 (3.7)**	0.001*
**MCH (pg)** (27–32)	29.6 (0.5)	29.9 (2.0)	**29.7 (1.0)**	22.1 (1.7)	22.3 (1.4)	**22.2 (1.6)**	21.0 (1.6)	22.0 (1.3)	**21.5 (1.4)**	< 0.000*
**MCHC (%)** (33–35)	33.0 (0.5)	33.5 (0.7)	**33.2 (0.6)**	31.4 (0.8)	31.6 (1.0)	**31.5 (0.9)**	31.7 (1.1)	31.5 (0.6)	**31.6** **(0.8)**	0.002*
**Reticulocytes (%)** (0.5–1.5)	9.6 (2.1)	6.3 (5.5)	**8.5 (4.5)**	5.3 (1.6)	5.1 (1.7)	**5.2 (1.6)**	4.8 (3.0)	5.1 (2.4)	**5.0 (2.6)**	0.048*
**Absolute Retic count (×10**^**12**^**/L)** (0.020–0.200)	0.2767 (0.1041)	0.1866 (0.1328)	0.2467 (0.1104)	0.2066 (0.0669)	0.1905 (0.0609)	0.1988 (0.0635)	0.1643 (0.0856)	0.1855 (0.0896)	0.1759 (0.0840)	0.244
**WBC (× 10**^**9**^**/L)** (4.5–11.0)	14.0 (8.8)	6.7 (0.1)	11.6 (7.8)	8.7 (3.6)	10.5 (5.6)	9.59 (4.7)	10.2 (5.5)	12.2 (7.4)	11.34 (6.4)	0.863
**PLT (× 10**^**3**^**/**μL**)** (150–450)	324.0 (178.0)	252.0 (111.8)	300 (151.2)	235.5 (155.0)	330.3 (151.5)	281.4 (158.3)	254.6 (42.8)	347.2 (199.5)	305.1 (151.6)	0.715

*Hb* Haemoglobin, *Hb A2* Adult Haemoglobin-2, *Hb F* Foetal Haemoglobin, *MCV* Mean Corpuscular Volume, *MCH* Mean Corpuscular Haemoglobin, *MCHC* Mean Corpuscular Haemoglobin Concentration, *WBC* White Blood Cells, *PLT* Platelets. *p* < 0.05 of Kruskal Wallis H test was taken as significant

*p* value has been calculated with respect to total figures of the three groups (Hb SS, severe Hb S/β+ and Hb S/β^0^ type)

### Clinical findings

Age at presentation of all SCD patients was highly variable, ranging from 4 months to 55 years (Mean 9.8 years; SD- 11.3 years). Most of the patients (51.7%; *n* = 31) had presented with fever, whilst the next common presenting symptoms were joint pain and abdominal pain (26.7%; *n* = 16). Icterus led to identification of the disease in a further 9 (15%) cases. Six more patients were incidentally diagnosed whilst investigating anaemia. A further three patients were diagnosed during pregnancy (including two Hb S/β^0^ patients and one severe Hb S/β+ patient). Three out of 9 (33.33%) Hb SS, 1/37 (2.7%) severe Hb S/β+ and 2/14 (14.3%) Hb S/β^0^ (*p* 0.012) patients were on regular blood transfusion (defined as > 8 transfusions/year). Based on clinical records it appeared that blood transfusions had mostly been given when haemoglobin concentration of the patient fell to 6 g/dl or less, although this could not be ascertained with certainty. Incidentally, 12 (23.53%) of SBT patients (7 severe Hb S/β+ and 5 Hb S/β^0^ patients) never had any transfusion in their lifetime.

Clinical features of Hb SS, severe Hb S/β+ and Hb S/β^0^ and patients are summarized in Table 2. Joint pains were the most common clinical symptom observed among all SCD patients. Ischemic cerebrovascular event had occurred in one severe Hb S/β+ and one Hb SS patient. Similarly, avascular necrosis of the hip was present in one Hb SS patient and one severe Hb S/β+ patient. Fisher’s exact test showed that the incidence of dactylitis was the only clinical feature which was significantly different between Hb SS, severe Hb S/β+ and Hb S/β^0^ patients (*p* 0.027). Nevertheless, none of the clinical features were significantly different between severe Hb S/β+ and Hb S/β^0^ patients. Genotype-phenotype associations were also assessed separately between Hb SS, severe Hb S/β+ and Hb S/β^0^ patients those who were on regular transfusions and those who were not. Nevertheless, Fisher’s exact test was unable to find any difference between the patients in the two transfusion categories. Splenectomy had been carried out in 1 / 9 Hb SS and 4/51 severe Hb S/β+ patients. The exact reason for splenectomy and its justification could not be deduced from the clinical records. Four of the splenectomized patients had undergone the surgery before the age of 20 years. Forty-one SCD patients (6 Hb SS, 23 severe Hb S/β+ and 12 Hb S/β^0^ patients) (68.3%) in our series had a history of at least one pain event (Joint/Abdominal/Chest) in their lifetime, while 19 SCD patients including three Hb SS individuals had not experienced any pain events. Cold weather (33.33%, *n* = 20) was the most frequently identified precipitating factor for pain events among SCD individuals, followed by infections (26.32%, *n* = 15). Thirteen (21.66%) SCD patients who had pain crisis reported no obvious precipitant factor for pain events. At the time of data collection 26 (43.33%) SCD patients were taking Hydroxyurea. Nineteen (31.67%) SCD patients were on Folic acid only. Twenty-eight (48.7%) SCD patients were on penicillin prophylaxis. Six SCD patients (10%) were not on any medication.

**Table 2:** Summary of clinical features observed between severe Hb S/β+, Hb S/β^0^ and Hb SS groups

Clinical feature/Complication	severe Hb S/β+ group *n = 37*	Hb S/β^0^ group *n = 14*	Hb SS group *n = 9*	Cumulative Figure *N* = 60	*p* value^1^ between two SBT groups	*p* value^2^ between all three groups
Joint pain	20 (54.05%)	12 (85.70%)	7 (77.78%)	39 (65.0%)	0.053	0.080
Palpable spleen	26 (70.27%)	7 (50.0%)	3 (33.33%)	36 (60.0%)	0.204	0.086
Hospital admission due to pain	20 (54.05%)	10 (71.43%)	5 (55.56%)	35 (58.33%)	0.346	0.562
Jaundice	14 (37.84%)	9 (64.28%)	6 (66.67%)	29 (48.33%)	0.120	0.109
Major infections	13 (35.14%)	4 (28.57%)	4 (44.44%)	21 (35.0%)	0.749	0.744
Recurrent headaches	11 (29.73%)	1 (7.14%)	2 (22.22%)	14 (23.33%)	0.142	0.281
Pica	6 (16.22%)	3 (21.43%)	2 (22.22%)	11 (18.33%)	0.692	0.711
Abdominal pain	6 (16.22%)	4 (28.57%)	0	10 (16.67%)	0.432	0.217
Dactylitis	3 (8.11%)	3 (21.43%)	4 (44.44%)	10 (16.67%)	0.327	0.027*
Gallstones	6 (16.22%)	3 (21.43%)	1 (11.11%)	10 (16.67%)	0.692	0.888
Pallor	4 (10.81%)	2 (14.28%)	3 (33.33%)	9 (15.0%)	0.661	0.220
Acute chest syndrome	3 (8.11%)	3 (21.43%)	2 (22.22%)	8 (13.33%)	0.327	0.290
Vision impairment	8 (21.62%)	0	0	8 (13.33%)	0.088	0.074
Abdominal distension	3 (8.11%)	1 (7.14%)	1 (11.11%)	5 (8.33%)	1.000	1.000
Facial deformities	1 (2.70%)	2 (14.28%)	0	3 (5.0%)	0.179	0.189
Nocturnal enuresis	2 (5.40%)	1 (7.14%)	0	3 (5.0%)	1.000	1.000
Leg ulcers	2 (5.40%)	1 (7.14%)	0	3 (5.0%)	1.000	1.000

^1^Fisher’s exact test *p* < 0.05 was taken as significant between two SBT groups

^2^Fisher’s exact test *p* < 0.05 was taken as significant between all three groups

### Genetic findings

Gap PCR for common α+ gene deletions found only 4 (6.67%) SBT patients with 3.7 kb deletions. None of the SCD patients had the 4.2 kb α gene deletion.

**Fig. 2:**
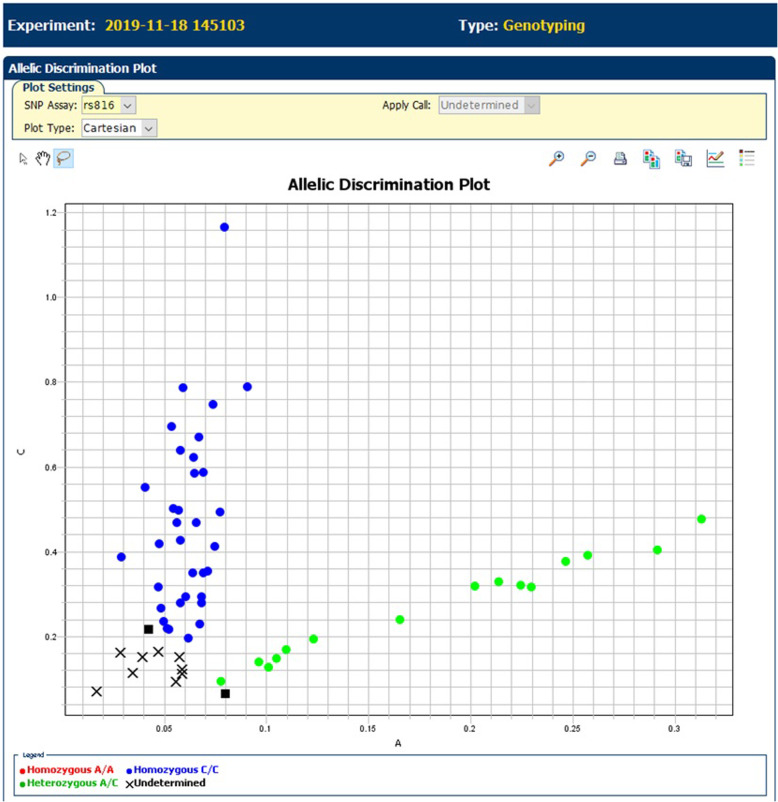
Allelic discrimination of the marker Hb F marker rs6545816 of SCD patients

Haplotyping by traditional RFLP showed that the sickle mutation occurred on two main beta globin haplotypes in Sri Lanka. Namely Arab-Indian (AI) and Benin. Out of 18 β globin haplotypes among the 9 Hb SS patients, 14 were AI haplotype and 4 were Benin haplotype. Presence of AI haplotype and Benin haplotype in Sri Lanka was confirmed with NGS by typing 4 different SNPs (rs3834466, rs28440105, rs10128556 and rs968857) in 9 Hb SS patients as described previously [27]. When looking at the genetic variants that moderate Hb F levels, rs6545816 in BCL11A was found at the highest allele frequency (88%) followed by rs7482144 in Xmn I-HBG2 (47%) (Table 3). Allelic discrimination plot of rs6545816 is shown in Fig. 2.

**Table 3:** Presence and frequency of Hb F determining variants in Sri Lankan SCD patients

Locus	Variants	Position on chromosome	Allele change	Genotypes detected	Hb F boosting allele (Frequency)
Chromosome 2					
BCL11A	rs6545816	60,568,365	A > C	CC, *n* = 37	C (88%)
				AC, *n* = 14	
	rs1427407	60,571,547	G > T	GG, *n* = 43	T (12%)
				GT, *n* = 14	
Chromosome 6					
HMIP-2A	rs66650371	135,460,326-135,460,328	In > Del	II, *n* = 52 DI, *n* = 5	Del (6%)
				DD, *n* = 1	
HMIP-2B	rs9402686	135,469,509	G > A	GG, *n* = 52	A (4%)
				GA, *n* = 4	
Chromosome 11					
Xmn I – HBG2	rs7482144	5,232,745	G > A	GG, *n* = 4	A (47%)
				GA, *n* = 56

## Discussion

Our study is the first description of the genotypic and phenotypic associations of SCD in Sri Lanka. Haplotype data in our present study sheds new light on the genetic background of Hb S in Sri Lanka. Both AI and Benin haplotypes of Hb S are common in Saudi Arabia [28, 29]. The presence of AI and Benin haplotypes of Hb S in Sri Lanka suggests that occurrence of Hb S in Sri Lanka is more likely to have originated from Arab migrations than African settlings: Historical records also indicate the existence of Arab settlements near coastal towns of Beruwala, Colombo, Chilaw, Galle, Mannar, Puttalam and Trincomalee by ninth century A.D. [30]. In the present study most of the SCD patients recruited were living fairly close to some of above-mentioned coastal areas.

There have been several island wide’s surveys of anaemia conducted in Sri Lanka and in a nationwide study involving 7526 adolescent age school children conducted in 2009–2010 anaemia prevalence was 172 (5.6%) in males and 298 (11.1%) in female children. In the same survey 28 (1.0%) male and 130 (4.6%) female students were found to have Iron deficiency anaemia. Further analysis of the same study identified that β thalassaemia trait and deletional α thalassaemia contributed to anaemia in a further 3%. Eleven children with Sickle cell trait had been identified in this study but there is no mention about their haemoglobin level [4, 31–33]. Though anaemia is identified to be an important finding in the present study, it is unlikely that SCD has a significant bearing on the national anaemia figures due to its relative low prevalence.

Joint pain was the most common clinical feature observed among Hb SS (77.78%), severe Hb S/β+ (54.05%) and Hb S/β^0^ (85.70%) patients. Joint pains are not uncommon among sickle patients in the Indian subcontinent. A recent study from Madhya Pradesh, Central India reported that the incidence of joint pain is over 80% in both Hb SS and SBT groups [34]. Bone pain reported at high frequencies in Indian SCD patients [24, 25, 35], was not present in any participants in our study. Requirement for regular blood transfusions was higher among Hb SS (33.33%) than severe Hb S/β+ (2.70%) and Hb S/β^0^ (14.30%) patients in the present study. These findings differ from a study reported from Madhya Pradesh (India) in which 16.1% of Hb SS and 17.4% of SBT patients were on regular blood transfusion [34]. Furthermore, seven severe Hb S/β+ (18.90%) and 5 Hb S/β^0^ (35.70%) patients had never received a transfusion in their lifetime. As the indication for blood transfusions were very often physician initiated and there was no defined rationale, these observations need to be interpreted with caution, and suggests the need for clear guidelines on the management of SCD in Sri Lanka, including the use of transfusion.

Only two cases of avascular necrosis of the hip were reported in the present study, which is in contrast to eastern Indian SCD patients, in whom incidences over 10% have been reported across several age groups [26]. Priapism and leg ulcers were not found in our study. There were no deaths in any of the SCD patients reported during the period of observation.

Hb F plays a significant role in ameliorating complications is SCD [36]. In the present study, in patients who were not on transfusion, mean Hb F concentrations were 24.4%, 23.7% and 21.2% in Hb SS, severe Hb S/β+ and Hb S/β^0^ patients, respectively. These values are in accordance with the observations from the Maharashtra (India) but are higher than the values observed in Madhya Pradesh (India) [25, 34]. The Hb F levels are much higher than those found in SCD patients of Sub-Saharan African origin, and levels greater than 20% would typically be associated with less severe clinical picture. Hb F boosting allele “C” of rs6545816 was detected at a much higher frequency in Sri Lankan patients than in patients from the United Kingdom (34%), Tanzania (36%) and Nigeria (35%) [18, 19]. Furthermore, among those who were not on transfusion and hydroxyurea (*n* = 22), “T” allele of rs1427407 was significantly associated with high Hb F levels (*p* 0.046). The presence of common α+ gene deletions was surprisingly low in this study. This is in contrast with observations in Western Indian SCD patients in whom the prevalence of α gene deletions was 29/51;56.8% [19]. Similarly, in a study of 60 SCD patients in New Delhi, the prevalence of α globin gene deletions was 18/60;30.% [37].

One of the most notable observational findings of our study was the gross inconsistency in the clinical management of SCD patients across the different centres across Sri Lanka. Usage of hydroxyurea was not consistent among sickle patients in these centres. Equally, the practice of blood transfusions was also very variable, reflecting perhaps the lack of familiarity in the management of the disease by the attending medical teams and the need for national guidelines on the management of patients with SCD. The prevalence of SCD in Sri Lanka however is rare permitting us to gather data from only 60 SCD patients which is a limitation in the present study.

## Conclusions

Overall, though the numbers may be limited the clinical course of the Asian version of SCD in Sri Lanka appears to be milder than that described from India. High Hb F levels are common and deletional α thalassaemia rarer. The natural selection, early migratory patterns of Arabs and settlements may explain why SCD is found mostly in coastal regions and low lands of Sri Lanka. We plan to undertake further work to elucidate the causative elements responsible for the milder appearance of SCD in Sri Lanka.

## Data Availability

The datasets used and/or analyzed during the current study are available from the corresponding author on reasonable request.

## Abbreviations

SCD: Sickle Cell Disease
HBB: β-haemoglobin subunit
Hb S: Sickle Haemoglobin
Hb SS: Homozygous Sickle Cell Anaemia
SBT: Sickle β-thalassaemia
EDTA: Ethylenediaminetetraacetic acid
CE: Capillary Electrophoresis
PCR: Polymerase Chain Reaction
RFLP: Restriction Fragment Length Polymorphism
ARMS: Amplification Refractory Mutation System
NGS: New Generation Sequencing
Hb F: Foetal Haemoglobin
